# Meet up‐and‐coming analytical scientists – Yulin Qi

**DOI:** 10.1002/ansa.202200046

**Published:** 2023-01-01

**Authors:** Yulin Qi

**Affiliations:** ^1^ Institute of Surface‐Earth System Science, School of Earth System Science Tianjin University Tianjin China; ^2^ Tianjin Key Laboratory of Earth Critical Zone Science and Sustainable Development in Bohai Rim Tianjin University Tianjin China

**Abbreviations**
ECRs: early career researchers ECR
FT‐ICR MS: Fourier‐transform ion cyclotron resonance mass spectrometry
NOM: natural organic matter

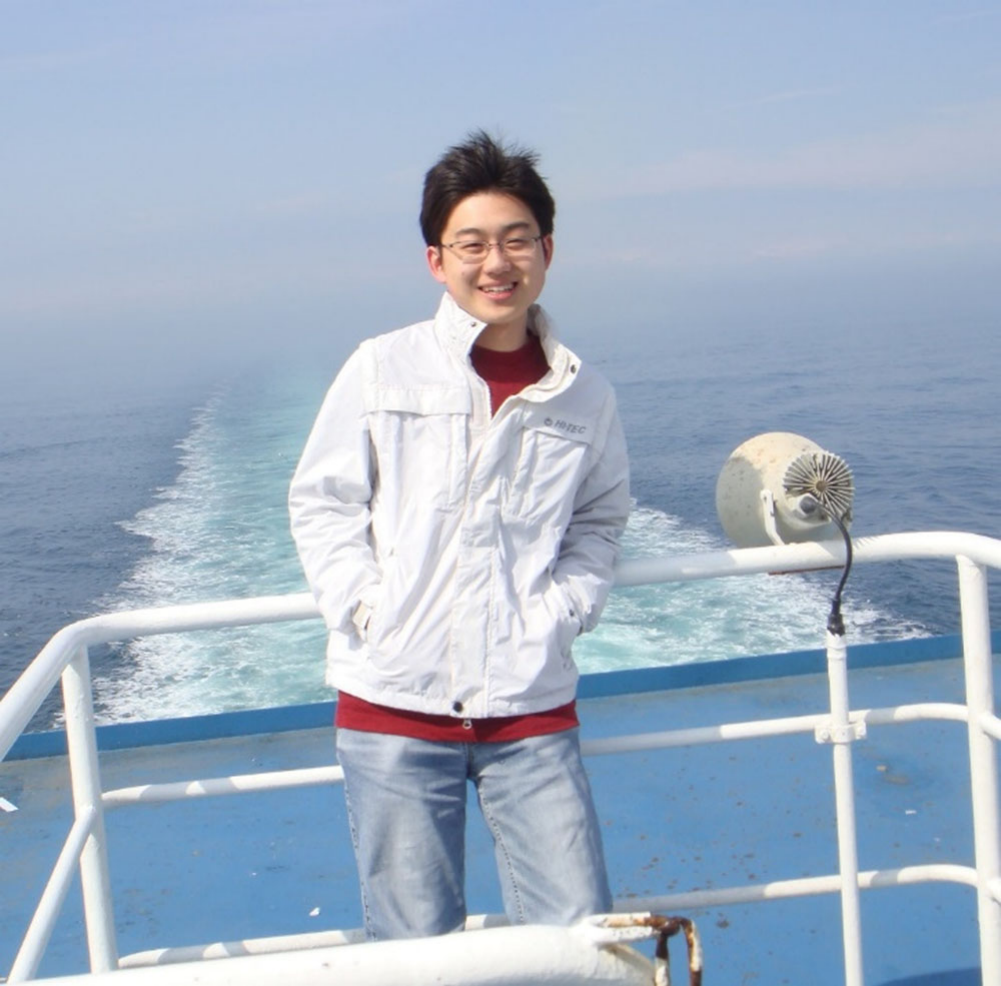
 Analytical sciences are among the most dynamically developing fields and have been inherently integrated into many various scientific disciplines. At the same time, early career researchers (ECRs) are among those whose contribution to this dynamic growth cannot be simply overestimated. Hence, in this special issue “From one ECR to the next”, we are presenting a series of editorials with questions and answers from five emerging scientists of different analytical fields including omics, environmental, and data sciences. Importantly, all our guests boast not only scientific excellence and high‐quality research but also the substantial international experience gained during their PhD or postdoctoral training. For this editorial, we are presenting Dr Yulin Qi.

Dr Yulin Qi received his PhD degree from the University of Warwick in 2013, under the supervision of Prof. Peter O'Connor. After that, he worked at Saarland University and the Humboldt University of Berlin, with Prof. Dietrich Volmer. In 2019, he went back to China and joined Tianjin University, and he was promoted to full professor in 2021. In 2020, he was awarded the Mattauch‐Herzog Award by the German Society for Mass Spectrometry, and he is the first Asian awardee in its 34‐year history. Yulin's research is focused on the development and application of Fourier‐transform ion cyclotron resonance mass spectrometry (FT‐ICR MS) to the study of natural organic matter (NOM).

## What is your original background?

Well, I was major in pharmaceutics as an undergraduate, but I found it too boring to remember the massive medical prescriptions, so I decided to study analytical chemistry afterwards.

## What is your current research focus?

As we know that NOM is a complex mixture of degraded natural compounds which plays a crucial role in global carbon cycling. It is actively involved in many biogeochemical processes. Additionally, NOM also leads to issues such as water odour, disinfectant demands, membrane fouling, and the production of carcinogenic disinfection byproducts. Revealing the elemental composition and molecular reactivities of NOM is the key to understanding its role in biogeochemical processes. Of the wide varieties of analytical instruments available, FT‐ICR MS holds the greatest potential for state‐of‐the‐art NOM research, given its inherently ultra‐high resolving power and mass accuracy for unequivocal molecule assignments. For this reason, it is currently the instrument of choice in geochemistry, environmental science and atmospheric chemistry for the analysis of organic matter in different environmental matrices at the molecular level. My current research is based upon the development and application of FT‐ICR MS to the study of NOM, including structural characterization, quantitative analysis, biogeochemical cycle, data processing and other related fields.

## What is your biggest motivation to work in analytical science?

As I said, I was not good at remembering medical prescriptions, but I found it enjoyable to play with analytical instruments during my undergraduate study.

## Of all your research projects, which one was your favourite and why?

That was my PhD project. My supervisor Prof. Peter O'Connor handed me a 40‐year‐old FT‐ICR MS computational problem to try to solve. The reason was that my boss was new to the university and the instruments were not installed yet, so I cannot but take up a computer modelling project. Luckily, with the help of Steven VanOrden (Global FT‐ICR MS Acquisition & Device Control Manager from Bruker Daltonics), I finally found a feasible solution that doubled the performance of any FT‐ICR mass spectrometer. And now Bruker has embedded this algorithm into their standard instrument control software. Thus, our research has already made a significant economic impact on the community!

## What was your motivation for choosing postdoctoral training?

When I graduated, I also got offers from an instrument company and a tobacco company. To be honest, my choice for postdoctoral training has nothing to do with science. I was worried that I might be settled down all my life in a city if I chose an offer from industry. For this reason, I decided to take a postdoctoral position at Saarland University (which is at the French‐German border, also very close to Luxembourg and Belgium, which is a nice place).

## What was your biggest (if any) culture shock experience in the country of your postdoc?

Of course, the language problem. For me, German is the most difficult language in the world!

## In your scientific career, what was the best or worst advice you ever heard from anyone?

“Always keep in mind that you're in the driver's seat, not your supervisor. You have to lead, and your supervisor is only supposed to mentor you.” Luckily, my supervisors (Prof. O'Connor and Prof. Volmer) gave me the freedom to choose research topics, the freedom to purchase materials, and present hypotheses, results, and reasoning. For this reason, I was involved in various research fields and always be passionate about exploring methods to find the answers.

## What advice would you give to someone looking for a postdoc position now?

Do not focus too much on a specific topic, maybe try and learn something different from your postgraduate work, as science is becoming more and more interdisciplinary nowadays.

## What is your favourite non‐scientific activity?

I like reading so much because it forces me to use my imagination in a whole new world to enjoy the story and I think that is one of the best things in this world. It helped when I thought my head would explode from overthinking things.

## Who (three people but not scientists!) would you invite to a dream dinner party?

(1) Cixin Liu, the most influential Chinese science fiction writer. I just have so many questions about his books. (2) Gordon Ramsay, the British chef, and television personality can at least cook the dinner and just be generally awesome. (3) Christopher Nolan, is best known for his cerebral, often nonlinear, storytelling, acclaimed movies.

## CONFLICT OF INTEREST

The author declares no conflict of interest.

